# Metabolite-assisted models improve risk prediction of coronary heart disease in patients with diabetes

**DOI:** 10.3389/fphar.2023.1175021

**Published:** 2023-03-24

**Authors:** Min Shen, Qingya Xie, Ruizhe Zhang, Chunjing Yu, Pingxi Xiao

**Affiliations:** ^1^ Department of Endocrinology and Metabolism, The First Affiliated Hospital of Nanjing Medical University, Nanjing, Jiangsu, China; ^2^ Department of Cardiology, The Fourth Affiliated Hospital, Nanjing Medical University, Nanjing, China; ^3^ Department of Nuclear Medicine, Affiliated Hospital of Jiangnan University, Wuxi, China

**Keywords:** metabolic marker-assisted model, risk prediction, diabetics, cardiovascular diseases, nomograms

## Abstract

**Background:** Patients with diabetes have a two-to four-fold increased incidence of cardiovascular diseases compared with non-diabetics. Currently, there is no recognized model to predict the occurrence and progression of CVDs in diabetics.

**Objective:** This work aimed to develop a metabolic biomarker-assisted model, a combination of metabolic markers with clinical variables, for risk prediction of CVDs in diabetics.

**Methods:** A total of 475 patients with diabetes were studied. Each patient underwent coronary angiography. Plasma samples were analyzed by liquid chromatography-quadrupole time-of-flight mass spectrometry. Ordinal logistic regression and random forest were used to screen metabolites. Receiver operating characteristic (ROC) curve, nomogram, and decision curve analysis (DCA) were employed to evaluate their prediction performances.

**Results:** Ordinal logistic regression screened out 34 differential metabolites (adjusted-false discovery rate *p* < 0.05) from 2059 ion features by comparisons of diabetics with and without CVDs. Random forest identified methylglutarylcarnitine and lysoPC (18:0) as the metabolic markers (mean decrease gini >1.0) for non-significant CVDs (nos-CVDs) *versus* normal coronary artery (NCA), 1,3-Octadiene and 3-Octanone for acute coronary syndrome (ACS) *versus* nos-CVDs, and lysoPC (18:0) for acute coronary syndrome *versus* normal coronary artery. For risk prediction, the metabolic marker-assisted models provided areas under the curve of 0.962–0.979 by ROC (0.576–0.779 for the base models), and c-indices of 0.8477–0.9537 by nomogram analysis (0.1514–0.5196 for the base models). Decision curve analysis (DCA) showed that the models produced greater benefits throughout a wide range of risk probabilities compared with the base model.

**Conclusion:** Metabolic biomarker-assisted model remarkably improved risk prediction of cardiovascular disease in diabetics (>90%).

## Introduction

Patients with diabetes are at increased risk of developing cardiovascular diseases (CVDs), and worse outcomes when CVDs are present (Haffner et al., 1998; Hayward et al., 2015; Writing Group et al., 2016). CVD-stimulated heart attack and stroke are by far the most frequent causes of death in diabetics (Colhoun et al., 2004; Wannamethee et al., 2004; Hu et al., 2005; Emerging Risk Factors et al., 2010; Nicholls, 2017). Diabetics with concomitant CVDs can be categorized into two broad groups (Haffner et al., 1998): those with non-significant cardiovascular diseases (nos-CVDs) consisting of non-obstructive coronary atherosclerosis and stable angina, and (Hayward et al., 2015) those with acute coronary syndrome (ACS) consisting of unstable angina and acute myocardial infarction.

If the progression of CVDs can be accurately predicted in patients with diabetes, early intervention may be achieved to avoid or delay the development of heart diseases. Timely secondary prevention was shown to improve the prognosis of CVD-patients with diabetes (Collins et al., 2003; Hu et al., 2005; Keech et al., 2005). To date, few recognized models have been developed to predict the presence and progression of CVDs in diabetics (Gyberg et al., 2015). There is therefore the need for improved risk stratification tools for diabetics.

Biomarkers continue to be discovered to complement clinical assessment of disease risk (Dunn et al., 2011; Gouveia et al., 2013; Menni et al., 2013; Rodriguez-Gallego et al., 2015). Metabolomics is a rapidly expanding field in system biology to measure alterations of metabolites and to identify metabolic biomarkers in response to disease processes. Discovery of metabolite signatures improves early diagnosis, prognostic prediction, and therapeutic intervention of CVD (Keech et al., 2005). This study aims to develop metabolic biomarker-assisted models, combining metabolites and clinical variables, for risk prediction of CVDs in patients with diabetes.

## Methods

Study Population. A total of 475 diabetic patients were recruited from the Sir Run Run Hospital of Nanjing Medical University during August 2017 and December 2022. Each patient underwent coronary angiography for confirmation of presence and severity of CVDs. Samples were randomly distributed into the training set (291 individuals) and the test set (184 individuals).

Plasma samples of the patients were collected before the coronary angiography surgery and quickly stored at −80°C for further metabolomic analyses. Blood biochemical indices (HbA1c; triglyceride, TG; total cholesterol, TC; high-density lipoprotein, HDL; and low-density lipoprotein, LDL) were performed, and the history of diseases and smoking history were recorded using questionnaire. Those patients with other cardiac-related diseases, blood-related disorders, infectious diseases, and malignant tumors were excluded. All subjects signed the informed consent forms. This study was approved by the ethics committee of Sir Run Run Hospital of Nanjing Medical University and complied with the Helsinki Declaration.

Sample Preparation. To eliminate the protein in the plasma, 150 μL of acetonitrile was added to a 50 μL aliquot of plasma and vortexed for 10 s. Precipitated protein was subsequently removed by centrifugation (13,000 rpm, 10 min) at 4°C. Then, 150 μL of the supernatant was transferred to a tube and dried under a gentle stream of nitrogen gas at room temperature. Finally, the supernatant was reconstituted in 100 μL of aqueous acetonitrile (8:2, *v/v*) for LC/MS detection.

Quality Control Sample. To ensure data quality for metabolic profiling, quality control (QC) sample was proceeded. The detail process of QC referred to Fan *et al* (Fan et al., 2016).

Metabolomics Study. Liquid chromatographic separation was conducted using a 1,290 Infinity System (Agilent Technologies, United States), with 100 × 2.1-mm Zorbax Eclipse Plus 1.8-mm C18 column maintained at 45°C. The mobile phase consisted of water with 5 mM ammonium acetate (A) and 10% aqueous acetonitrile with 5 mM ammonium acetate (B). Gradient program of elution was: 5%–80% B at 0–7 min, 80%–100% B at 7–12 min, 100% B at 12–13 min, and then back to initial conditions, and 2 min for equilibration. The sample volume injected was 1 μL and the flow rate was 0.4 mL/min.

The mass spectrometric detection was performed on an Agilent 6530 Q/TOF-MS system (Agilent Technologies, United States) in positive mode. The parameters were set as: the fragmental voltage at 100 V, nebulizer gas at 35 psig, capillary voltage at 3500 V, drying gas flow rate at 10 L/min, and temperature at 300°C. Reference masses at m/z 121.0509 and 922.0098 were introduced for accurate mass calibration.

MassHunter Workstation Software (version B.06.00, Agilent Technologies) was used to convert mass spectrometry data (d) into data format (.mzdata) files. XCMS package (Scripps Center for Metabolomics and Mass Spectrometry, La Jolla, California) was used to conduct the data pre-treatment, including non-linear retention time alignment, peak discrimination, filtering, alignment, matching, and identification. The detailed information of the experiment has been described in previous study (Fan et al., 2016).

Statistical Analysis. Prior to statistical analysis, log_2_ transformation of metabolite profiles was performed to transform data approximated to normal distribution. Continuous variables were described as mean (standard deviation) or median (interquartile range (IQR)). For validating our findings, the test sets were essential. At any exploratory stage, follow-up test sets verified the reliability of the results of training set.

The false discovery rate (FDR) using Benjamini and Hochberg method was calculated to address the multiple test adjustment. All tests were two-sided, and *p* < 0.05 were considered statistically significant unless stated otherwise. All analyses were conducted using R Software Version 3.3.1.

Model Development. Potential biomarkers were selected from metabolites profile with the criterion of adjusted FDR *p* < 0.05 by ordinal logistic regression presented both in the training and test sets. The mean decrease gini (MDG) of these metabolites was then calculated using random forest. Non-condition logistic regression was employed to get the area under the curve (AUC) of each model. The discriminative abilities of the multivariate models were assessed by Harrell’s concordance index (c-index), as reported previously (Harrell et al., 1982). Internal bootstrap validation, bias-corrected 95% confidence intervals for odds ratios in the final model, and bootstrap optimism corrected c-index were calculated using 1,000 re-samples (Chen and George, 1985). Finally, decision curve analysis (DCA) was applied to evaluating clinical benefit of the models.

## Results

From a total of 475 diabetic patients enrolled in the study, 291 were randomly selected as the training set, including 20 NCA subjects, 69 nos-CVD patients, and 202 ACS patients. To further validate the results of the training set, the other 184 patients were served as the test set (9 NCA, 51 nos-CVDs, and 124 ACS patients). The clinical characteristics of the patients are summarized in Table 1.

**Table 1:** Basic information in training and test sets.

	Training set			*p*-value	Test set				*p*-value	
	NCA	Nos-CVD	ACS		NCA	Nos-CVD	ACS			
	20	69	202		9	51	124			
Gender				0.004					0.012	
Female	11	36	70		7	31	55			
Male	9	33	132		2	20	69			
Age,years				0.101					0.152	
Median	57.5	62	64		62	64	64			
IQR	51.7-66.5	57.0-67.0	57.0-70.0		59.0-65.0	58.0-68.5	59.8-70.3			
HH				0.700					0.249	
Yes	16	58	171		6	43	108			
No	4	11	31		3	8	16			
SH				0.361					0.711	
Yes	5	14	50		1	10	25			
No	15	55	152		8	41	99			
HbA1c,%				0.129					0.230	
Median	6.4	6.8	7.1		6.7	7.2	7.25			
IQR	6.04-7.53	6.22-7.82	6.41-8.42		6.10-6.80	6.60-8.50	6.56-8.53			
TG,mmol/l				0.150					0.791	
Median	1.2	1.78	1.74		1.23	2.14	1.76			
IQR	1.02-2.10	1.13-2.56	1.21-2.70		1.03-1.62	1.45-3.13	1.36-2.78			
TC,mmol/l				0.869					0.773	
Median	4.61	4.47	4.46		3.78	4.5	4.25			
IQR	4.16-5.22	3.66-5.16	3.68-5.33		3.65-4.43	3.81-5.45	3.60-5.02			
HDL,mmol/l				<0.001					0.346	
Median	1.17	1.07	0.97		1.12	0.99	0.99			
IQR	0.96-1.46	0.86-1.26	0.83-1.13		0.81-1.28	0.82-1.24	0.83-1.11			
LDL,mmol/l				0.239					0.598	
Median	2.7	2.63	2.74		2.01	2.45	2.55			
IQR	2.21-3.30	2.04-3.12	2.16-3.34		1.94-2.73	2.07-3.30	1.88-3.09			

IQR, inter-quartile range; ACS, acute coronary syndrome; nos-CVD, non-significant cardiovascular disease; NCA, normal coronary artery; HH, hypertension history; SH, smoking history; HbA1c = glycosylated hemoglobin; TG, triglyceride; TC, total cholesterol; HDL, high-density lipoprotein cholesterol; LDL, low-density lipoprotein cholesterol.

**FIGURE 1:**
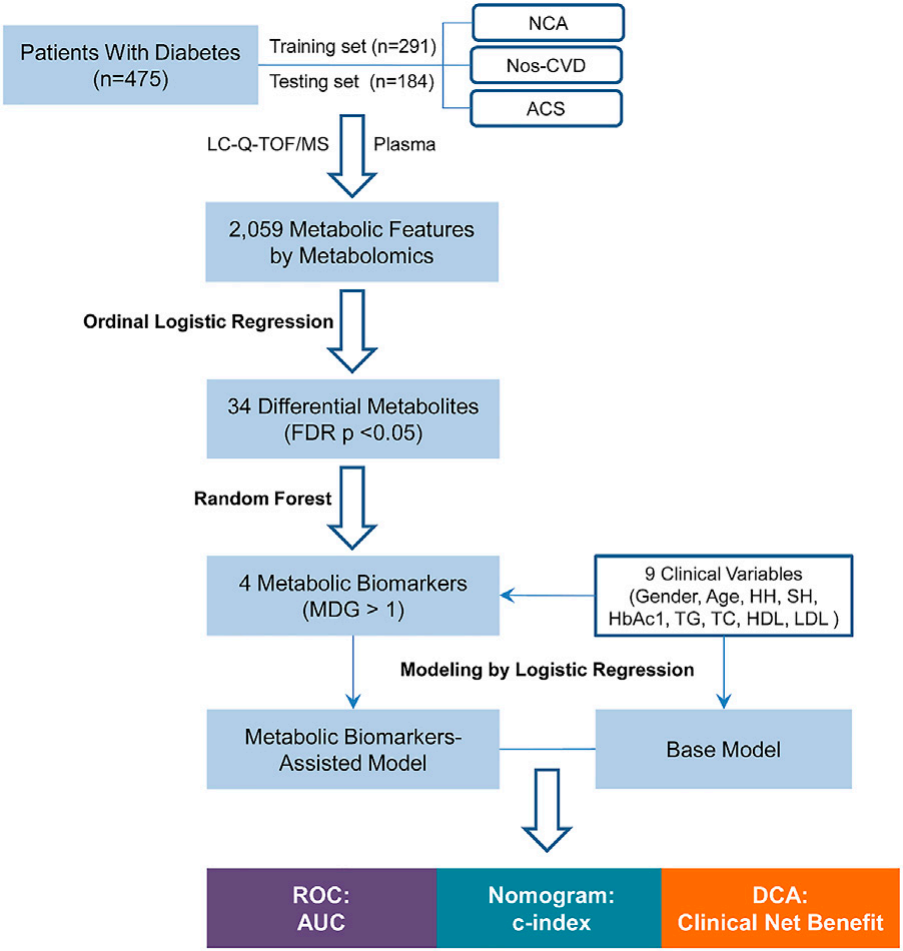
Flow Chart of the study. NCA = normal coronary artery; Nos-CVDs = non-significant cardiovascular diseases; ACS = acute coronary syndrome; LC-Q-TOF/MS = liquid chromatography-quadrupole time-of-flight mass spectrometry; FDR = false discovery rate; MDG = mean decrease gini; HH = hypertension history; SH = smoking history; HbA1c = glycosylated hemoglobin; TG = triglyceride; TC = total cholesterol; HDL = high-density lipoprotein cholesterol; LDL = low-density lipoprotein cholesterol; ROC = receiver operating characteristic; AUC = area under the curve; c-index = concordance index; DCA = decision curve analysis.

Compared with NCA and nos-CVDs groups, the ACS patients in the two sets showed an increased trend in HbA1c and triglyceride (TG) levels, and a decreased trend in high-density lipoprotein (HDL) level, (Table 1). Additionally, we found that the HbA1c levels positively correlated with CVDs progression (Supplementary Figure S1; Figure 1A), and individuals with high HbA1c levels had much higher CVD risk in the combined set (Supplementary Figure S2 **;** Figure 1B).

The flowchart of the risk prediction model is presented in Figure 1. Firstly, ordinal logistic regression and random forest were used to screen the most important metabolic markers for the model development. Then receiver operating characteristic (ROC) curve, nomogram, and DCA were used to evaluate their risk prediction performances.

A total of 2,059 positive-mode ions were detected. The ordinal logistic regression was employed to identify the significant ion signatures among NCA, nos-CVDs, and ACS. Forty-two metabolites with adjusted-FDR *p* < 0.05 were identified to be associated with CVDs progression in the training set (Supplementary Table S1). Importantly, 34 of the 42 metabolites were further confirmed in the test set by adjusted-FDR *p* < 0.05, as shown in Table 2. Identification of the metabolites was performed by comparison of their MS data with Human Metabolome Database (HMDB), and 15 of the 34 metabolites were further confirmed with reference compounds. Calibration curves were used to determine the concentrations of the 34 metabolites in 475 plasma samples. For the metabolites without reference compounds, relative quantification was performed using calibration curves of metabolites close to the analyte.

**Table 2:** The metabolites identified by both in the training and test sets.

Metabolizes	NCA	Nos-CVD	ACS	*p*-value^a^
	Median (IQR, μM/L)	Median (IQR, μM/L)	Median (IQR, μM/L)	
^b^Glycocholic acid	2.64 (2.19–3.52)	1.73 (1.23–2.28)	0.30 (0.20–0.33)	6.32e-44
Decenyl acetate	0.10 (0.08–0.11)	0.11 (0.09–0.15)	0.04 (0.04–0.05)	1.33e-28
3-Octanone	2.24 (1.68–2.38)	2.42 (2.03–3.15)	1.06 (0.79–1.26)	7.33e-25
^b^LysoPC(18:1)	19.84 (17.41–21.38)	13.32 (10.50–18.15)	7.21 (5.69–7.92)	8.00e-25
^b^Fumaric acid	2.72 (2.60–2.79)	2.64 (2.53–2.77)	2.32 (2.22–2.41)	1.38e-22
Indole-3-ethanol	0.07 (0.07–0.08)	0.05 (0.04–0.05)	0.04 (0.03–0.04)	2.33e-22
1,3-Octadiene	3.92 (2.58–4.29)	3.82 (2.76–5.11)	1.67 (1.42–1.88)	8.89e-22
^b^LysoPC(18:0)	32.67 (25.13–38.51)	21.58 (15.04–28.92)	13.14 (10.73–15.32)	1.34e-21
Trimethylamine N-oxide	0.22 (0.18–0.31)	0.32 (0.29–0.40)	0.46 (0.40–0.56)	2.72e-19
N-Phenylacetyl-L-glutamine	2.02 (1.84–2.23)	3.27 (2.64–3.82)	5.03 (3.95–6.15)	3.30e-19
^b^LysoPC(24:0)	0.37 (0.30–0.38)	0.31 (0.28–0.34)	0.22 (0.18–0.27)	1.06e-18
Phosphocholine	3.21 (2.61–3.97)	2.89 (2.25–3.99)	2.01 (1.46–2.42)	1.64e-13
Phosphatidylcholine	0.42 (0.39–0.49)	0.47 (0.38–0.60)	0.29 (0.22–0.35)	3.33e-13
2-Hydroxylauric acid	0.44 (0.38–0.58)	0.56 (0.44–0.67)	0.76 (0.62–0.89)	9.51e-12
PI (20:4/0:0)	1.77 (1.43–2.14)	1.66 (0.97–2.27)	2.81 (2.14–3.43)	2.12e-11
^b^Ethylchenodeoxycholic acid	3.34 (2.30–3.94)	4.08 (3.26–4.62)	2.61 (1.84–3.32)	3.42e-11
^b^LysoPC(22:6)	7.35 (5.75–9.05)	6.41 (5.40–7.41)	5.09 (4.06–6.12)	1.21e-10
Methylglutarylcarnitine	0.09 (0.07–0.09)	0.11 (0.10–0.13)	0.08 (0.06–0.09)	6.35e-10
Creatine	40.20 (35.36–46.15)	34.29 (27.22–42.28)	47.40 (39.14–55.41)	6.43e-09
^b^Valine	245.40 (211.10–250.80)	230.10 (220.00–247.60)	208.80 (192.40–223.40)	1.02e-08
Undecan 3-ol	0.16 (0.14–0.20)	0.15 (0.13–0.18)	0.12 (0.10–0.15)	1.54e-08
^b^LysoPC(20:3)	8.65 (6.26–12.12)	9.12 (7.34–10.72)	6.72 (5.56–7.89)	2.06e-08
LysoPE (18:3)	0.71 (0.65–0.78)	0.59 (0.50–0.70)	0.49 (0.38–0.56)	1.47e-07
^b^LysoPE (16:0)	5.75 (4.16–6.51)	5.16 (4.78–6.17)	4.23 (3.45–5.26)	2.26e-07
^b^Phytosphingosine	2.93 (2.83–3.08)	3.30 (2.17–3.79)	4.49 (3.28–5.32)	9.91e-07
^b^Glutarylcarnitine	0.30 (0.26–0.36)	0.44 (0.31–0.53)	0.29 (0.22–0.35)	1.15e-05
^b^Succinic acid	14.92 (13.46–18.43)	15.18 (13.27–16.95)	13.60 (10.92–15.59)	1.04e-04
2-Nonynoic acid	0.81 (0.75–0.85)	0.59 (0.54–0.72)	0.56 (0.48–0.64)	1.41e-04
2α-Methyl-5α-androstane-3-17-dione	0.46 (0.34–0.50)	0.22 (0.17–0.27)	0.11 (0.10–0.13)	1.41e-04
PG (15:0/14:0)	6.67 (6.19–7.09)	6.35 (5.78–6.94)	5.76 (5.04–6.61)	3.01e-04
^b^γ-Aminobutyric acid	2.67 (2.52–2.99)	2.63 (2.22–2.92)	2.08 (1.66–2.75)	8.23e-04
Docosahexaenoic acid	1.66 (1.33–2.69)	2.16 (1.81–2.71)	1.69 (1.23–2.19)	9.39e-04
^b^LysoPE (18:1)	0.64 (0.61–0.93)	0.67 (0.56–0.85)	0.59 (0.47–0.69)	2.09e-03
LysoPE (22:5)	0.46 (0.44–0.49)	0.44 (0.37–0.58)	0.38 (0.29–0.48)	3.66e-03

^b^ Means that the metabolites were tentatively identified with reference compounds.

^a^ Ordinal logistic regression adjusted by false discovery rate (FDR). IQR, inter-quartile range; ACS, acute coronary syndrome; nos-CVD, non-significant cardiovascular disease; NCA, normal coronary artery.

Interval. ACS, acute coronary syndrome; nos-CVD, non-significant cardiovascular disease; NCA, normal coronary artery.

**FIGURE 2:**
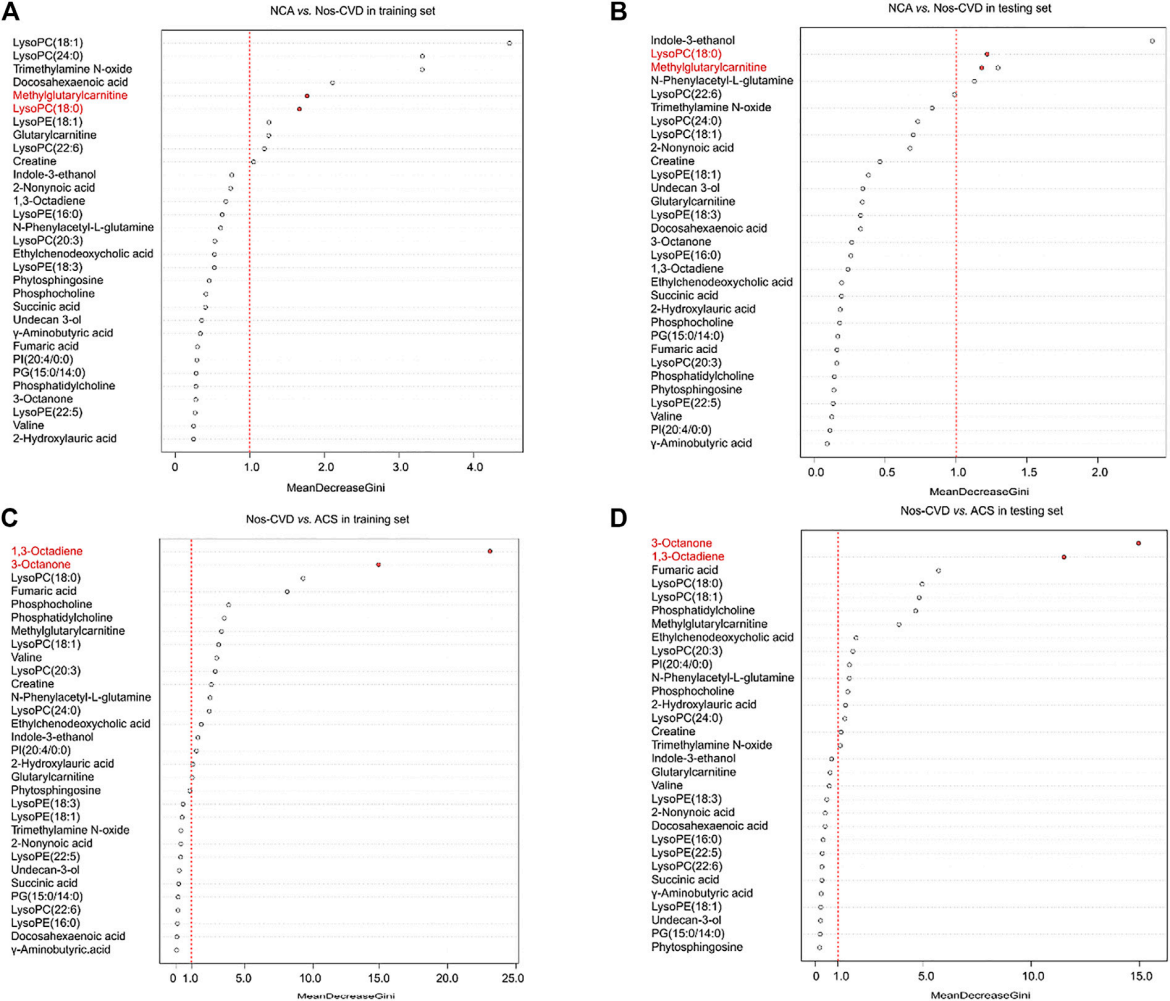
The variable importance measure (VIM) of 34 metabolites by random forest for normal coronary artery (NCA) *versus* non-significant cardiovascular disease (nos-CVD) in training set **(A)** and in test set **(B)**, and for nos-CVDs *versus* acute coronary syndrome (ACS) in training set **(C)** and in test set **(D)**. The metabolites were ranked based on a decreasing order in mean decrease gini (MDG).

**FIGURE 3:**
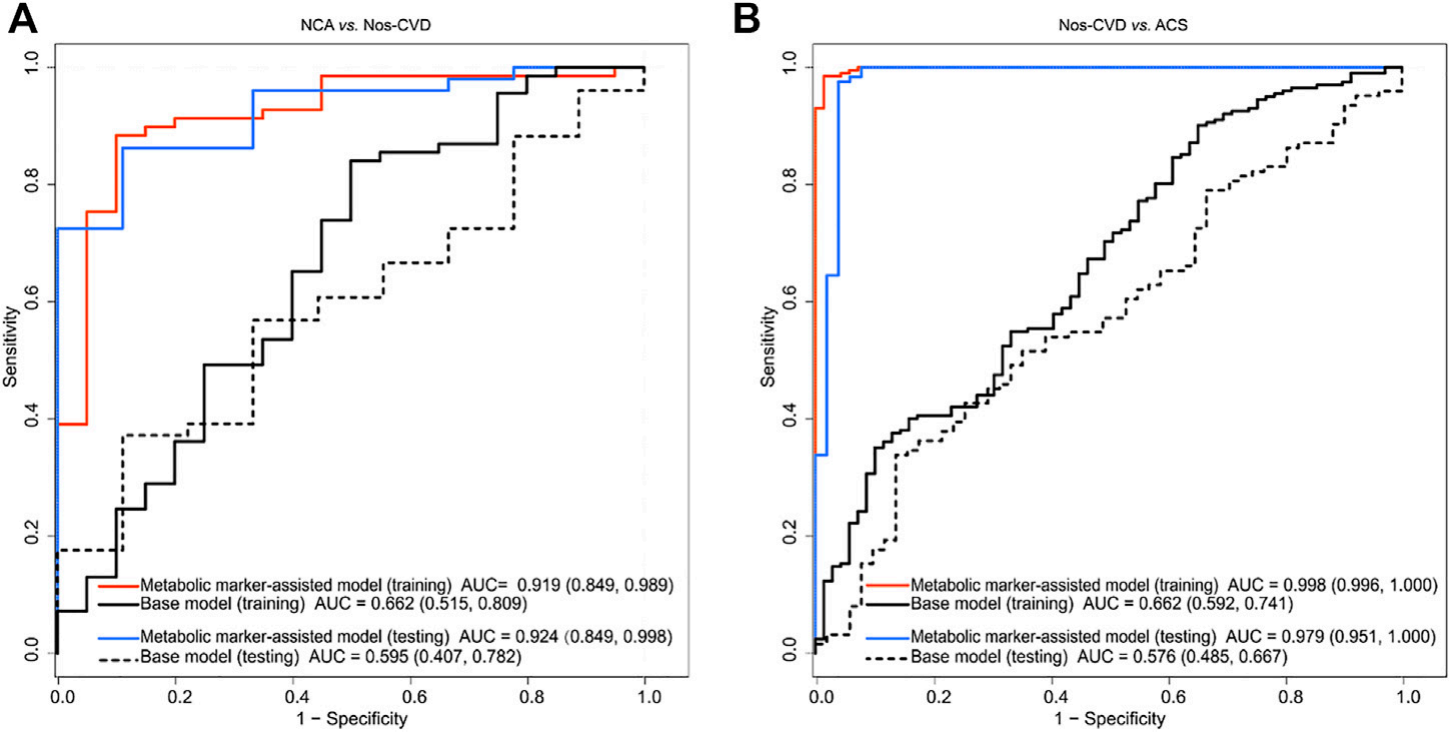
Receiver operating characteristic (ROC) curve analyses of base models and metabolic merker-assisted models **(A)** Area under the curve (AUC) for discriminating normal coronary artery (NCA) from non-significant cardiovascular diseases (nos-CVDs) **(B)** AUC estimation for discriminating nos-CVDs from acute coronary syndrome (ACS).

To estimate the variable importance measure (VIM) of these 34 metabolites, the random forest model was utilized to calculate their mean decrease gini (MDG). The metabolites with MDG values > 1.0 present both in the training and test sets were chosen as the metabolic biomarkers for further model development. Methylglutarylcarnitine and lysoPC (18:0) were identified for NCA *versus* nos-CVDs (Figures 2A, B), 1,3-Octadiene and 3-Octanone for nos-CVDs *versus* ACS (Figures 2C, D), and lysoPC (18:0) for NCA *versus* ACS (Supplementary Figure S2).

Clinical Roc Evaluation. Using the clinical variables (gender, age, hypertension history, smoking history, HbA1c, TG, TC, HDL, and LDL), we developed a base model by the logistic regression to distinguish the CVD stages in diabetic patients. Metabolic biomarker-assisted models, combining metabolic biomarkers from the random forest with clinical variables, were established for enhancing risk prediction of CVDs in diabetics. The detailed variables of base and metabolic marker-assisted models are listed in the Supplementary Table S2.

The difference of clinical diagnosis between the models was evaluated by logistic regression models in the training set. Compared with the base models, the metabolic marker-assisted models showed remarkably increased areas under the curve (AUC). The AUC increased from 0.662 (95% CI: 0.515–0.809) to 0.919 (95% CI: 0.849-0.989, *p* = 2.26e-4) for NCA *versus* nos-CVDs (Figure 3A), from 0.662 (95% CI: 0.592–0.741) to 0.998 (95% CI: 0.996-1.00, *p* = 1.10e-18) for nos-CVDs *versus* ACS (Figure 3B), and from 0.779 (95% CI: 0.669–0.889) to 0.987 (95% CI, 0.969-1.00, *p* = 3.19e-4) for NCA *versus* ACS (Supplementary Figure S3).

The test set confirmed the findings of the training set. The AUC significantly improved from 0.595 (95% CI: 0.407–0.782) to 0.962 (95% CI: 0.849-0.998, *p* = 4.97e-4) for NCA *versus* nos-CVDs (Figure 3A), from 0.576 (95% CI: 0.485–0.667) to 0.979 (95% CI: 0.951-1.00, *p* = 1.14e-17) for nos-CVDs *versus* ACS (Figure 3B), and from 0.745 (95% CI: 0.553–0.937) to 0.962 (95% CI, 0.885-1.00, *p* = 3.26e-3) for NCA *versus* ACS (Supplementary Figure S3).

**FIGURE 4:**
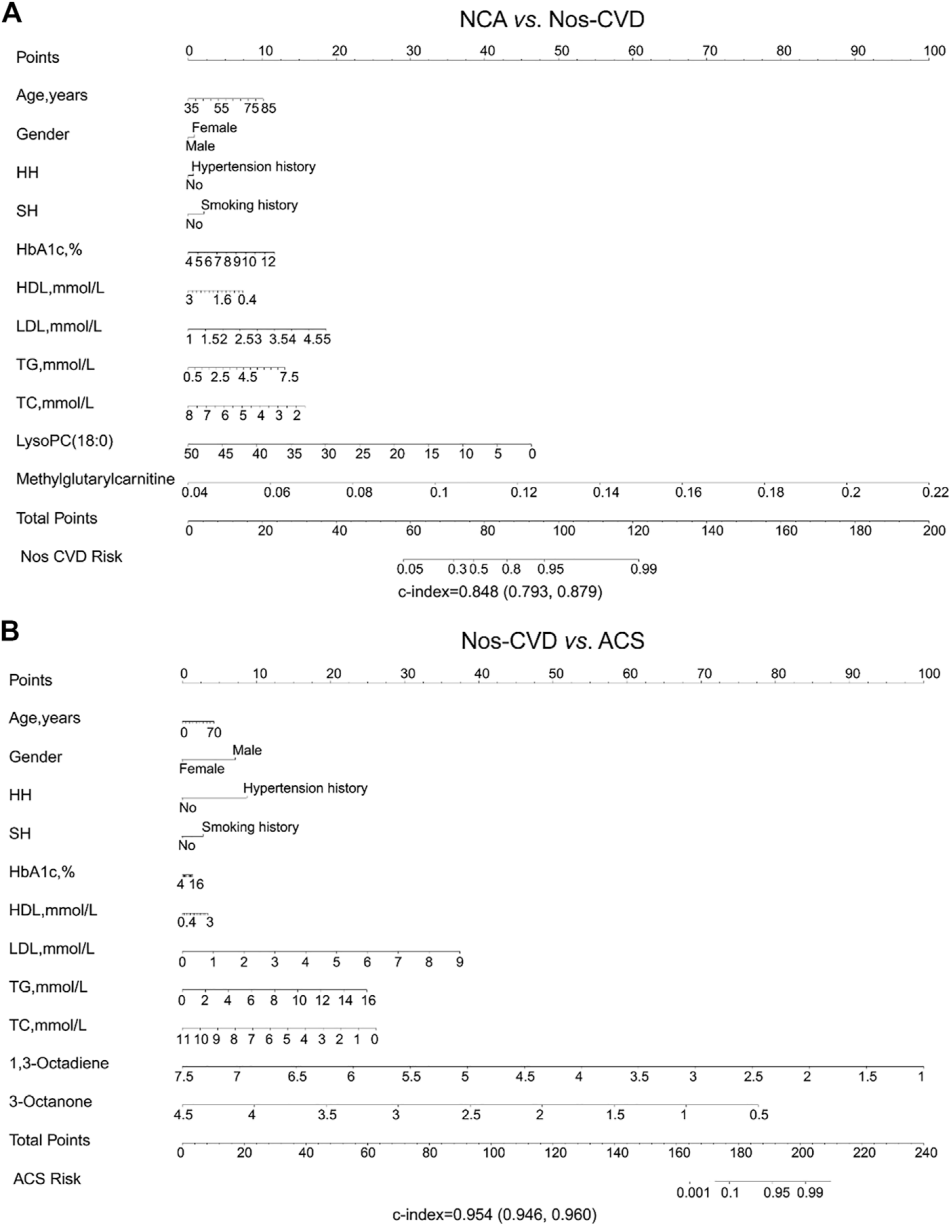
Nomogram depicting cardiovascular disease (CVD) risk among diabetics **(A)** Risk prediction for normal coronary artery (NCA) *versus* non-significant cardiovascular diseases (nos-CVDs) by metabolic marker-assisted model **(B)** Risk prediction for nos-CVDs *versus* acute coronary syndrome (ACS) by metabolic marker-assisted model. To obtain the predicted probability of CVD risk after diagnosis of diabetes, the patient values were located on each axis. A vertical line was drawn upward to the ‘Points’ axis to determine the points of the variable. The points for all variables were summed and located on the ‘Total points’ axis. A vertical line was drawn down to the ‘Nos-CVDs or ACS risk’ axis to find the patient’s probability of different CVDs type.

Development Of Nomograms. The nomograms for the prediction of CVDs stages were performed. In the base models, the c-indices were 0.151 (95% CI: 0.285–0.502) for NCA *versus* nos-CVDs, 0.519 (95% CI: 0.467–0.597) for NCA *versus* ACS, and 0.275 (95% CI: 0.238–0.319) for nos-CVDs *versus* ACS (Supplementary Table S3). The c-indices of the metabolic-assisted models were significantly improved, reaching up to 0.848 (95% CI: 0.793–0.880) for NCA *versus* nos-CVDs (Figure 4A), 0.954 (95% CI: 0.947–0.960) for nos-CVDs *versus* ACS (Figure 4B), and 0.952 (95% CI: 0.942–0.966) for NCA *versus* ACS (Supplementary Figure S4).

**FIGURE 5:**
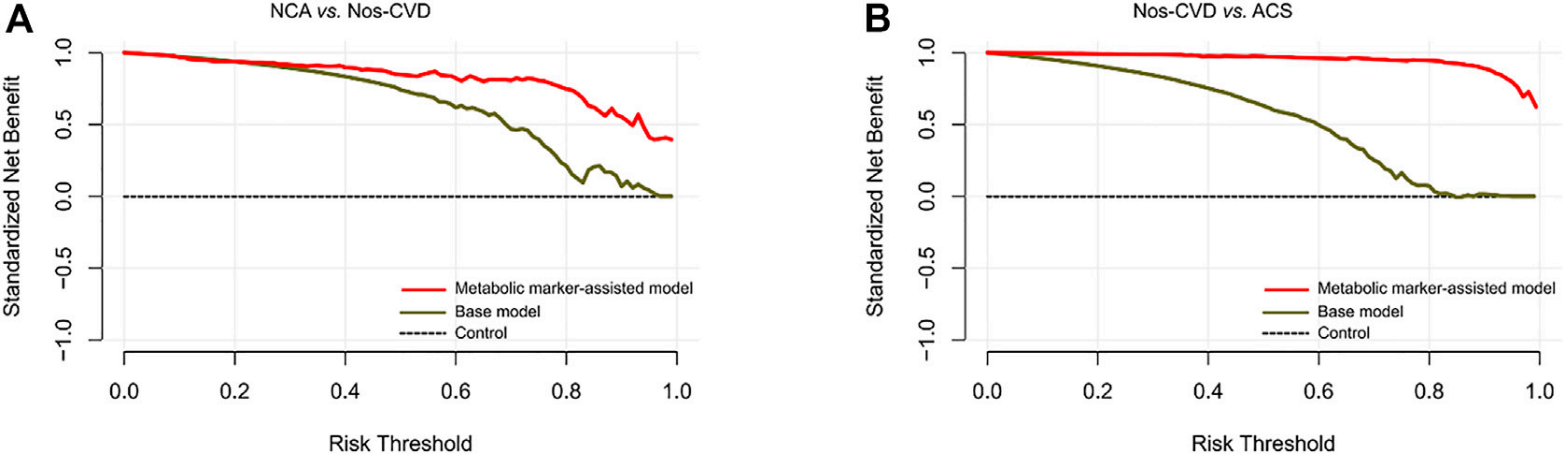
Decision curve analyses for clinical net benefits **(A)** Normal coronary artery (NCA) *versus* non-significant cardiovascular diseases (nos-CVDs) **(B)** Nos-CVDs *versus* acute coronary syndrome (ACS).

DCA Performances. The DCA was employed for the comparison of clinical net benefits between the models. Compared with the base models, metabolic marker-assisted models showed greater benefits throughout a wide range of risk probabilities: 33%–99% for NCA *versus* nos-CVD (Figure 5A), 2%–98% for nos-CVDs *versus* ACS (Figure 5B), and 11%–96% for NCA *versus* ACS in Supplementary Figure S5.

## Discussion

Diabetes is a main risk factor for CVDs, and promotes the progression of CVDs. Early screening of CVDs from diabetic patients is important to improve the patients’ prognosis. In this study, we constructed a promising predictive model consisting of nine clinical parameters and four metabolic markers for early identification of nos-CVDs and ACS in diabetics. Importantly, we found that the metabolic biomarkers significantly enhanced the model discrimination, leading to a greater improvement of clinical net benefits after addition to the base model.

The development and progression of CVDs involve many factors, including gender, age, exposure to adverse environmental conditions, genomic variations and alterations in the metabolome. Clinical risk models added with biomarkers have been previously shown to be superior to the clinical risk models alone (deFilippi et al., 2010; Schnabel et al., 2010; Velagaleti et al., 2010). In this work, four metabolites (methylglutarylcarnitine, lysoPC (18:0), 1,3-Octadiene, and 3-Octanone) verified from two sets remarkably increased the predictive abilities of the models for the stages of CVDs. Methylglutarylcarnitine, a derivative of a leucine, increased in nos-CVDs patients compared with the NCA group. Leucine is a nutritionally essential branched-chain amino acid (BCAA) in animal nutrition and is associated with energy metabolism (glucose uptake, mitochondrial biogenesis, and fatty acid oxidation) (Duan et al., 2016). Harald *et al.* reported leucine to be a cardiometabolic risk marker in a cross-sectional study (Mangge et al., 2016). The relationship between lysoPC (18:0) and CVDs have been widely investigated. High lysoPC (18:0) levels can decrease the risk of developing CVDs for diabetic patients (Herrmann et al., 2009; Stegemann et al., 2014). 1, 3-Octadiene and 3-Octanone can significantly increase the predictive efficiency of the model for nos-CVD *versus* ACS among the patients. To our knowledge, their roles in CVDs have been less studied.

To capture significant metabolites for the prediction model, a series of statistical approaches were performed. Ordinal logistic regression model takes the rank-order of the outcomes into consideration, and can effectively reduce the risk of type I error than pairwise comparison (de Havenon et al., 2017). Metabolites associated with the CVD process were screened out. Additionally, the random forest, taking advantage of two powerful machine learning techniques (bagging and random features selection) (Michaelson and forestSV, 2012; Van Peer et al., 2017), eliminated redundant features and confirmed the most important metabolites. DCA was employed to ultimately elucidate the clinical significance of the metabolic marker-assisted models in comparison to base models. It showed a wide range of clinical risk probability. Our predicted models can provide help in the early screening of CVDs (>96%), especially for ACS (>98%). The results indicated that cooperation of clinical features and metabolites can accurately detect the stages of CVDs in diabetics.

### Study limitations

Firstly, we used diabetic patients with normal coronary after angiography examination as the control NCA group, and the sample size is small. Secondly, because of the small number of the NCA subjects, the age and gender of different groups were not matched. Thirdly, because of commercial unavailability of reference compounds, identification of metabolites is still a challenge. Fourthly, we used an untargeted metabolomics for screening of significant metabolites, and a targeted metabolomics using isotope internal standard for accurate quantification in complex plasma matrices is recommended in the future. Fifthly, future prospective confirmation in independent cohorts is warranted.

## Conclusion

Metabolic profiling characterized CVD progression in patients with diabetes. Metabolic markers-assisted model remarkably improved risk prediction of cardiovascular disease (>90%). Timely secondary prevention will be thus initiated for early intervention and benefits.

## Data Availability

The original contributions presented in the study are included in the article/Supplementary Materials, further inquiries can be directed to the corresponding authors.

## Abbreviations

ACS, acute coronary syndrome; AUC, area under the curve; CVDs, cardiovascular diseases; c-index, concordance index, DCA, decision curve analysis; FDR, false discovery rate; LC-Q-TOF/MS, liquid chromatography-quadrupole time-of-flight mass spectrometry; MDG, mean decrease gini; NCA, normal coronary artery; Nos-CVDs, non-significant cardiovascular diseases; ROC, receiver operating characteristic.
