# Supplementary material for: Metabolite-assisted models improve risk prediction of coronary heart disease in patients with diabetes
Source: Front Pharmacol. 2023 Mar 24;14:1175021. doi: 10.3389/fphar.2023.1175021 (PMC10081143; doi:10.3389/fphar.2023.1175021)

**Supplementary Figure legends**

**Supplementary Figure 1** The association between HbA1c and cardiovascular diseases (CVDs). (**A**)With the progression of CVDs, the percentage of HbA1c increased in non- significant CVDs (nos-CVDs) and acute coronary syndrome (ACS) patients, compared with normal coronary artery (NCA) diabetics. (**B**) Individuals with high HbA1c had much significantly higher risk of CVDs.

**Supplementary Figure 2** The variable importance measure (VIM) of 34 metabolites by random forest for normal coronary artery (NCA) versus acute coronary syndrome (ACS) in training set (A) and in test set (B)

**Supplementary Figure 3** Receiver operating characteristic (ROC) curve analysis of base and metabolic marker-based models for discriminating normal coronary artery (NCA) from acute coronary syndrome (ACS).

**Supplementary Figure 4** Nomogram depicting acute coronary syndrome (ACS) risk among normal coronary artery (NCA) patients with diabetes. To obtain the predicted probability of CVD risk after diagnosis of diabetes, the patient values were located on each axis. A vertical line was drawn upward to the ‘Points’ axis to determine the points of the variable. The points for all variables were summed and located on the ‘Total points’ axis. A vertical line was drawn down to the ‘ACS risk’ axis to find the NCA patient’s probability.

**Supplementary Figure 5** Decision curve analyses for clinical net benefits of normal coronary artery (NCA) versus acute coronary syndrome (ACS).

**Online Figure 1**


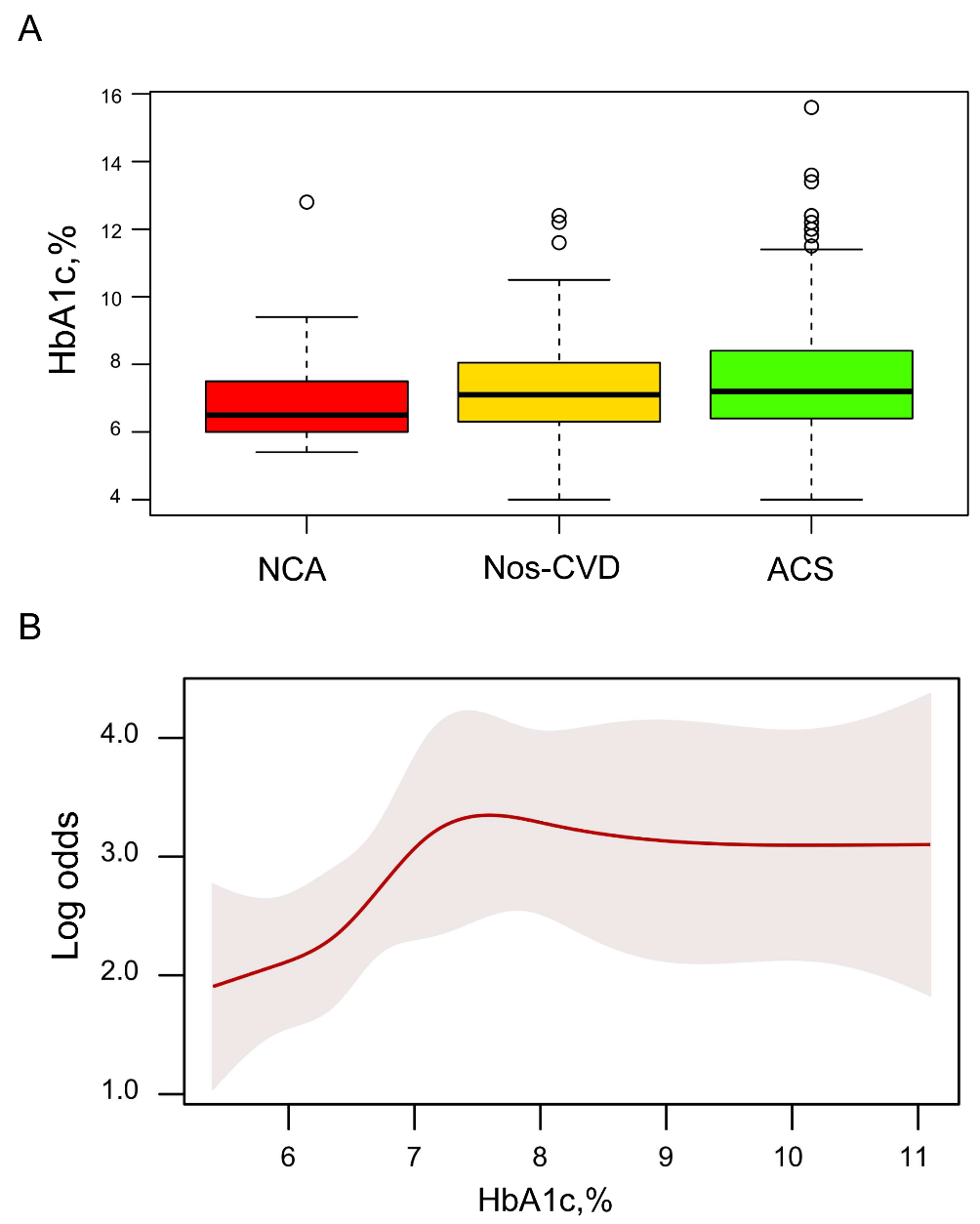


**Online Figure 2**


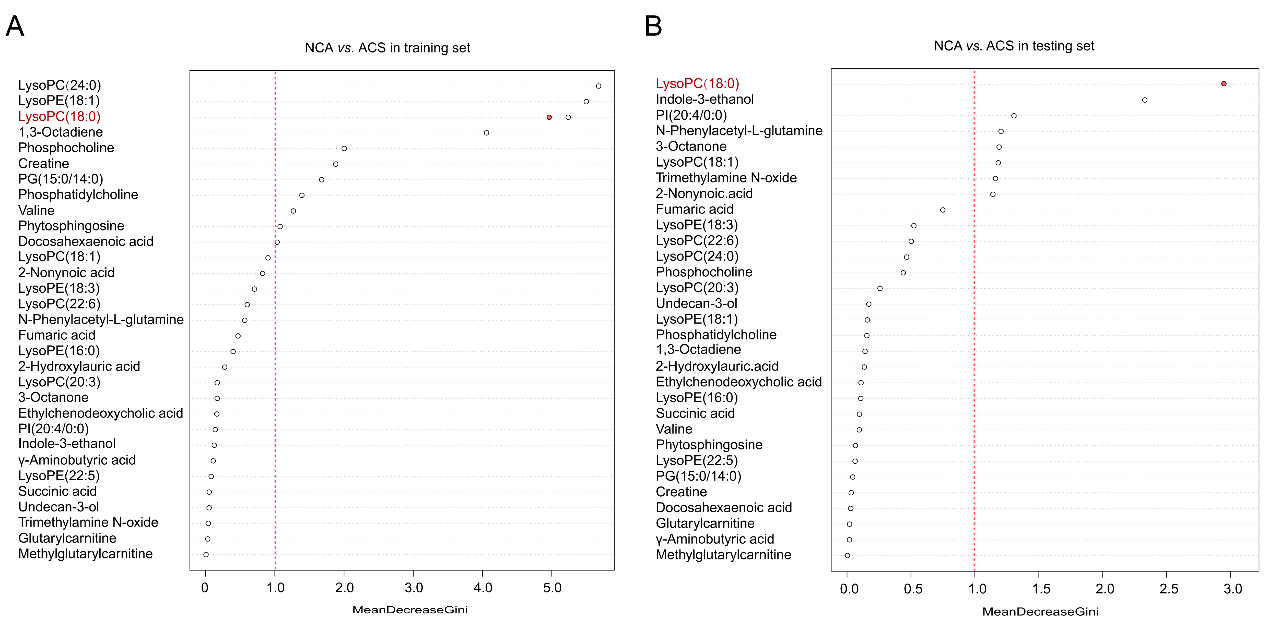


**Online Figure 3**


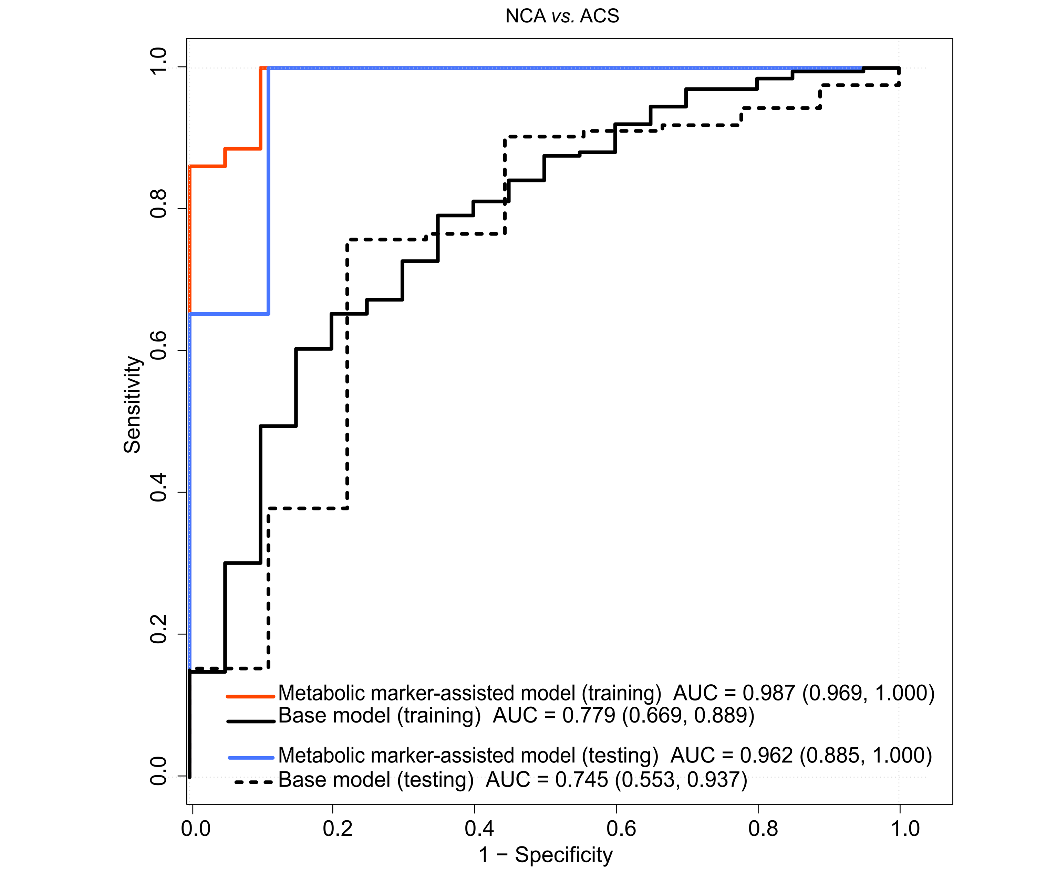


**Online Figure 4**


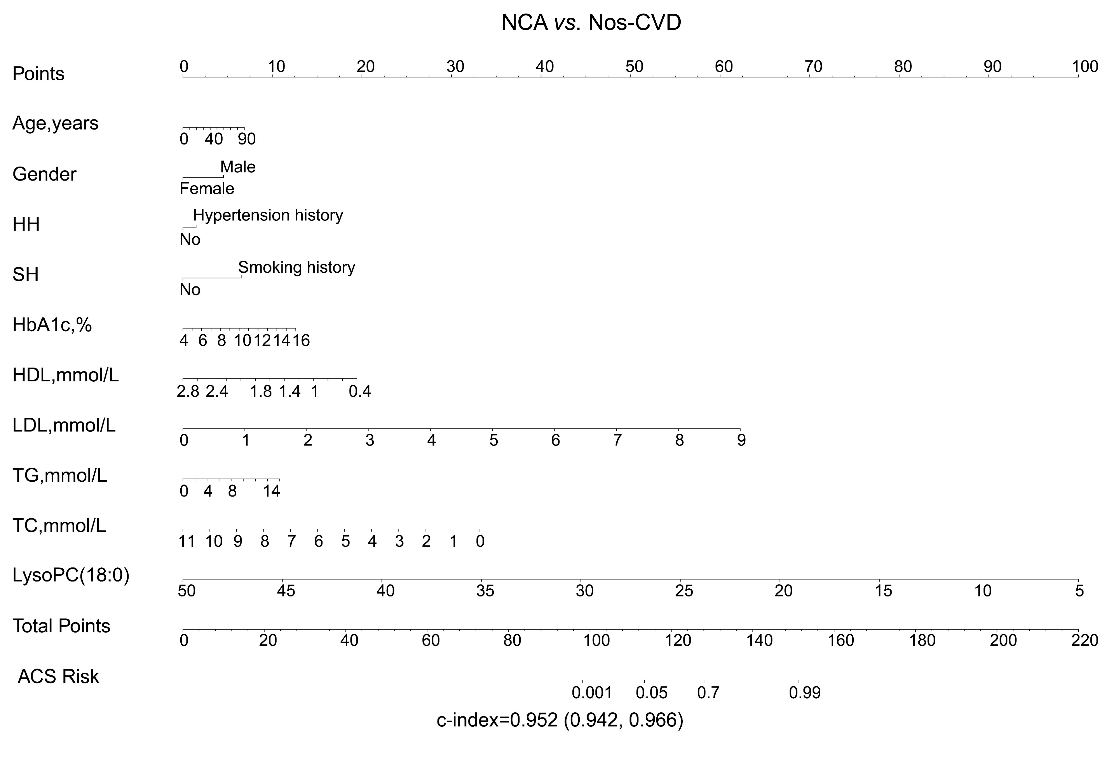


**Online Figure 5**


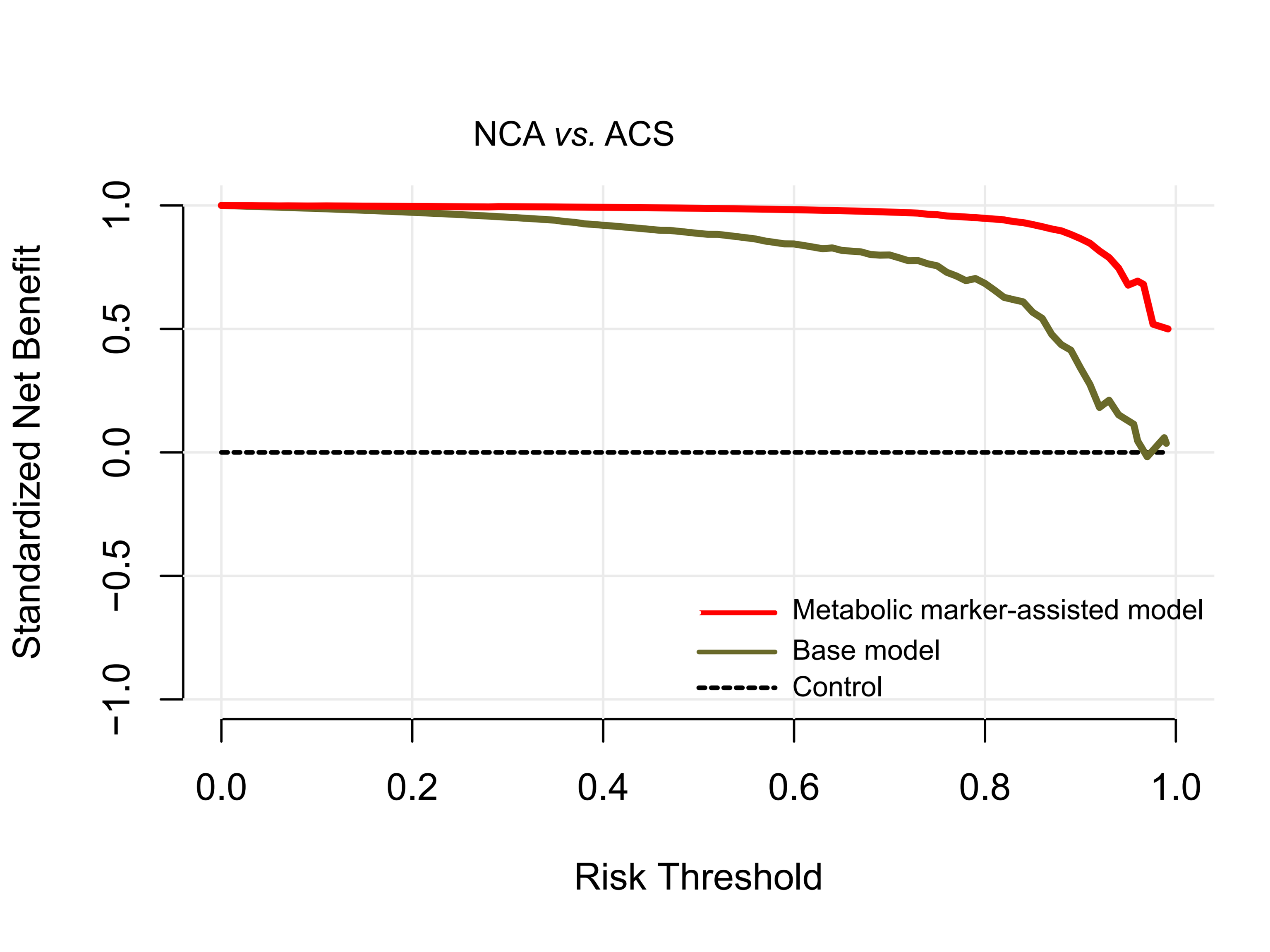

Supplement: Supplementary file 1 [file Table1.DOCX]
