# Supplementary material for: Metabolite-assisted models improve risk prediction of coronary heart disease in patients with diabetes
Source: Front Pharmacol. 2023 Mar 24;14:1175021. doi: 10.3389/fphar.2023.1175021 (PMC10081143; doi:10.3389/fphar.2023.1175021)
Supplement: Supplementary file 2 [file Table2.docx]

**Supplementary table 1** The metabolites from training set using ordinal logistic regression.

| **Metabolite** | **Beta** | **CL** | **CU** | ***p* value** | **FDR *p*** |
| --- | --- | --- | --- | --- | --- |
| **Glycocholic acid** | -5.22 | -6.59 | -3.86 | 1.29e-61 | 2.62e-58 |
| **2α-Methyl-5α-androstane-3-17-dione** | -30.62 | -37.92 | -23.33 | 4.94e-44 | 1.00e-40 |
| **Decenyl acetate** | -56.25 | -67.68 | -44.82 | 1.72e-40 | 3.49e-37 |
| **LysoPC(18:1)** | -0.34 | -0.41 | -0.27 | 1.35e-35 | 2.75e-32 |
| **LysoPC(24:0)** | -28.33 | -34.58 | -22.08 | 1.98e-31 | 4.02e-28 |
| **1,3-Octadiene** | -1.17 | -1.42 | -0.93 | 6.26e-31 | 1.27e-27 |
| **Indole-3-ethanol** | -167.68 | -205.05 | -130.30 | 1.87e-30 | 3.79e-27 |
| **LysoPC(18:0)** | -0.23 | -0.28 | -0.18 | 1.92e-30 | 3.90e-27 |
| **Fumaric acid** | -7.39 | -8.99 | -5.80 | 4.84e-28 | 9.83e-25 |
| **3-Octanone** | -1.96 | -2.40 | -1.52 | 2.05e-27 | 4.17e-24 |
| **N-Phenylacetyl-L-glutamine** | 1.20 | 0.91 | 1.49 | 1.34e-25 | 2.72e-22 |
| **Trimethylamine N-oxide** | 14.07 | 10.79 | 17.36 | 1.38e-23 | 2.80e-20 |
| **Phosphocholine** | -1.49 | -1.83 | -1.14 | 2.49e-23 | 5.06e-20 |
| **Phosphatidylcholine** | -11.04 | -13.61 | -8.47 | 3.22e-23 | 6.55e-20 |
| **Phytosphingosine** | 1.08 | 0.82 | 1.34 | 9.63e-22 | 1.96e-18 |
| **2-Hydroxylauric acid** | 7.65 | 5.74 | 9.57 | 1.54e-20 | 3.14e-17 |
| **Valine** | -0.05 | -0.06 | -0.03 | 2.41e-17 | 4.90e-14 |
| **PI(20:4/0:0)** | 1.24 | 0.90 | 1.58 | 9.51e-16 | 1.93e-12 |
| **Ethylchenodeoxycholic acid** | -0.89 | -1.13 | -0.65 | 1.29e-15 | 2.63e-12 |
| **Undecan 3-ol** | -29.59 | -37.92 | -21.25 | 6.06e-15 | 1.23e-11 |
| **Docosahexaenoic acid** | -1.63 | -2.09 | -1.17 | 1.71e-14 | 3.48e-11 |
| **Creatine** | 0.09 | 0.06 | 0.12 | 1.96e-14 | 3.99e-11 |
| **LysoPE(18:3)** | -7.11 | -9.29 | -4.92 | 3.42e-12 | 6.94e-09 |
| **Methylglutarylcarnitine** | -37.13 | -48.59 | -25.67 | 7.09e-12 | 1.44e-08 |
| **Palmitoylethanolamide** | -12.73 | -16.96 | -8.49 | 9.17e-10 | 1.86e-06 |
| **γ-Aminobutyric acid** | -1.30 | -1.75 | -0.85 | 9.39e-10 | 1.91e-06 |
| **LysoPC(22:6)** | -0.52 | -0.71 | -0.33 | 7.62e-09 | 1.55e-05 |
| **LysoPE(22:5)** | -5.97 | -8.12 | -3.83 | 8.35e-09 | 1.70e-05 |
| **Aspartic acid** | -0.23 | -0.32 | -0.15 | 1.60e-08 | 3.26e-05 |
| **Glutarylcarnitine** | -5.63 | -7.66 | -3.61 | 1.61e-08 | 3.27e-05 |
| **Succinic acid** | -0.25 | -0.34 | -0.16 | 1.83e-08 | 3.72e-05 |
| **Glycerophosphocholine** | -0.56 | -0.76 | -0.35 | 2.26e-08 | 4.60e-05 |
| **LysoPE(16:0)** | -0.60 | -0.83 | -0.38 | 4.71e-08 | 9.58e-05 |
| **PG(15:0/14:0)** | -0.78 | -1.07 | -0.48 | 4.94e-08 | 1.00e-04 |
| **X2_Nonynoic.acid** | -5.17 | -7.18 | -3.17 | 1.40e-07 | 2.84e-04 |
| **LysoPE(18:1)** | -3.45 | -4.80 | -2.11 | 2.10e-07 | 4.27e-04 |
| **LysoPC(18:2)** | -0.06 | -0.08 | -0.03 | 8.19e-07 | 1.66e-03 |
| **LysoPC(20:3)** | -0.27 | -0.38 | -0.16 | 1.35e-06 | 2.75e-03 |
| **Glycodeoxycholic acid** | -8.10 | -11.49 | -4.70 | 1.92e-06 | 3.90e-03 |
| **Isoleucylproline** | -7.67 | -11.13 | -4.21 | 2.85e-06 | 5.79e-03 |
| **LysoPE(18:0)** | -1.40 | -2.04 | -0.76 | 1.06e-05 | 2.15e-02 |
| **Tryptophan** | -0.03 | -0.04 | -0.02 | 1.89e-05 | 3.85e-02 |

Beta, regression slope and effect size; CU, upper of confidence interval; Cl, lower of confidence interval

**Supplementary table 2** The variables for cross-comparisons with and within CVDs in the two models

| **Subsets** | **Features** | **Metabolic-based model** | | | |  | **Base model** | | | |
| --- | --- | --- | --- | --- | --- | --- | --- | --- | --- | --- |
|  |  | **Beta** | **CL** | **CU** | ***p* value** |  | **Beta** | **CL** | **CU** | ***p* value** |
| **NCA  versus  nos-CVD** | **Gender** | 0.38 | -1.28 | 2.05 | 0.651 |  | -0.08 | -1.31 | 1.14 | 0.894 |
|  | **Age** | 0.01 | -0.09 | 0.12 | 0.822 |  | 0.04 | -0.03 | 0.12 | 0.256 |
|  | **HH** | -0.08 | -2.15 | 2.00 | 0.943 |  | -0.12 | -1.60 | 1.36 | 0.874 |
|  | **SH** | -0.67 | -2.56 | 1.21 | 0.482 |  | -0.46 | -1.81 | 0.90 | 0.508 |
|  | **HbA1c** | 0.17 | -0.39 | 0.73 | 0.558 |  | 0.13 | -0.25 | 0.51 | 0.495 |
|  | **TG** | 0.01 | -0.9 | 0.92 | 0.988 |  | 0.15 | -0.36 | 0.66 | 0.563 |
|  | **TC** | -0.01 | -1.85 | 1.82 | 0.988 |  | 0.20 | -0.76 | 1.16 | 0.690 |
|  | **HDL** | -0.84 | -4.14 | 2.46 | 0.618 |  | -1.25 | -3.09 | 0.60 | 0.186 |
|  | **LDL** | 0.32 | -1.78 | 2.43 | 0.762 |  | -0.38 | -1.48 | 0.72 | 0.502 |
|  | **LysoPC (18:0)** | -0.18 | -0.29 | -0.06 | 0.002 |  | \ | \ | \ | \ |
|  | **Methyl**  **glutarylcarnitine** | 89.41 | 40.31 | 138.51 | <0.001 |  | \ | \ | \ | \ |
|  |  |  |  |  |  |  |  |  |  |  |
| **NCA versus ACS** | **Gender** | -2.47 | -5.53 | 0.6 | 0.115 |  | -1.17 | -2.46 | 0.11 | 0.074 |
|  | **Age** | -0.04 | -0.24 | 0.17 | 0.721 |  | 0.06 | 0.01 | 0.10 | 0.023 |
|  | **HH** | -0.78 | -5.72 | 4.16 | 0.757 |  | -0.28 | -1.67 | 1.11 | 0.696 |
|  | **SH** | -0.44 | -5.8 | 4.92 | 0.873 |  | -0.75 | -2.13 | 0.62 | 0.282 |
|  | **HbA1c** | 0.78 | -0.45 | 2.00 | 0.215 |  | 0.22 | -0.16 | 0.59 | 0.259 |
|  | **TG** | 0.28 | -1.83 | 2.4 | 0.792 |  | 0.14 | -0.37 | 0.66 | 0.589 |
|  | **TC** | -1.05 | -4.85 | 2.76 | 0.590 |  | -0.16 | -1.17 | 0.85 | 0.762 |
|  | **HDL** | -2.55 | -7.85 | 2.75 | 0.346 |  | -2.36 | -4.35 | -0.37 | 0.020 |
|  | **LDL** | 2.46 | -2.54 | 7.46 | 0.334 |  | 0.456 | -0.75 | 1.67 | 0.459 |
|  | **LysoPC (18:0)** | -0.58 | -0.93 | -0.23 | 0.001 |  | \ | \ | \ | \ |
|  |  |  |  |  |  |  |  |  |  |  |
| **nos-CVD versus ACS** | **Gender** | -4.6 | -8.84 | -0.36 | 0.034 |  | -0.78 | -1.46 | -0.09 | 0.026 |
|  | **Age** | 0.12 | -0.06 | 0.29 | 0.198 |  | 0.034 | 0.00 | 0.06 | 0.034 |
|  | **HH** | -3.33 | -7.26 | 0.59 | 0.100 |  | -0.07 | -0.90 | 0.75 | 0.862 |
|  | **SH** | -1.47 | -5.46 | 2.52 | 0.470 |  | -0.18 | -0.96 | 0.60 | 0.644 |
|  | **HbA1c** | 0.67 | -0.66 | 2.00 | 0.322 |  | 0.14 | -0.06 | 0.35 | 0.170 |
|  | **TG** | 3.01 | 0.57 | 5.45 | 0.016 |  | 0.12 | -0.15 | 0.39 | 0.394 |
|  | **TC** | -5.55 | -9.93 | -1.18 | 0.013 |  | -0.31 | -0.91 | 0.29 | 0.312 |
|  | **HDL** | 7.09 | 0.56 | 13.61 | 0.033 |  | -0.82 | -2.10 | 0.46 | 0.208 |
|  | **LDL** | 5.43 | 1.17 | 9.69 | 0.013 |  | 0.69 | -0.02 | 1.41 | 0.058 |
|  | **1,3-Octadiene** | -8.12 | -13.51 | -2.74 | 0.003 |  | \ | \ | \ | \ |
|  | **3-Octanone** | -9.06 | -15.41 | -2.70 | 0.005 |  | \ | \ | \ | \ |

HH, hypertension history; SH, smking history; HbA1c, glycosylated hemoglobin; TG, triglyceride; TC, total cgolesterol; HDL, high-density lipoprotein cholesterol; LDL, low-density lipoprotein cholesterol; Beta, regression slope and effect size; CU, upper of confidence interval; Cl, lower of confidence interval. ACS = acute coronary syndrome; nos-CVD = non-significant cardiovascular disease; NCA = normal coronary artery.

**Supplementary table 3** The c-indices for nomograms in the two models.

| **Subsets** | **Marker-based model** | | |  | **Base model** | | |
| --- | --- | --- | --- | --- | --- | --- | --- |
|  | **c-index** | **CL** | **CU** |  | **c-index** | **CL** | **CU** |
| **NCA versus nos-CVD** | 0.848 | 0.793 | 0.879 |  | 0.285 | 0.151 | 0.503 |
| **NCA versus ACS** | 0.952 | 0.942 | 0.966 |  | 0.519 | 0.467 | 0.597 |
| **ACS versus nos-CVD** | 0.953 | 0.946 | 0.960 |  | 0.275 | 0.238 | 0.319 |

c-index, Harrell concordance index; CU, upper of confidence interval; Cl, lower of confidence interval. ACS = acute coronary syndrome; nos-CVD = non-significant cardiovascular disease; NCA = normal coronary artery.
